# Yield vulnerability of low-income smallholders to pollinator declines in Brazil is biome-dependent

**DOI:** 10.1371/journal.pone.0337328

**Published:** 2025-11-25

**Authors:** Willams Oliveira, Rafaella Guimarães Porto, Oswaldo Cruz-Neto, Marcelo Tabarelli, Blandina Felipe Viana, Carlos A. Peres, Ariadna Valentina Lopes

**Affiliations:** 1 Departamento de Botânica, Universidade Federal de Pernambuco, Recife, Pernambuco, Brazil; 2 Programa de Pós-Graduação em Biologia Vegetal, Departamento de Botânica, Universidade Federal de Pernambuco, Recife, Pernambuco, Brazil; 3 Instituto de Biologia, Universidade Federal da Bahia, Salvador, Bahia, Brazil; 4 School of Environmental Sciences, University of East Anglia, Norwich Research Park, Norwich, United Kingdom; 5 Instituto Juruá, Manaus, Brazil; National Museums of Kenya Ornithology Section, KENYA

## Abstract

Habitat loss and degradation, mainly driven by agricultural expansion and intensification, alter ecological processes, ecosystem services and human well-being at a global scale. Pollinator populations in degraded agricultural areas tend to collapse, which in turn can reduce the effective pollination of agricultural crops and food production, particularly in leading food producing countries, such as Brazil. We sought to understand how the vulnerability to pollinator failure and the economic value of pollination (EVP) are associated with farmland size, farmer income, habitat loss, and the size of the human footprint. We also examine socioeconomic predictors related to income inequality levels across Brazilian municipalities and phytogeographic domains. We show that 58% of all leading crops in Brazil are dependent to some degree on animal pollination, and agricultural production in 96.8% of all municipalities is at least partly vulnerable to pollinator failure. Soybean, coffee, cotton, tomato and cocoa accounted for 84% of the total economic valuation of cropland pollination in Brazil, with soybean alone representing more than half of this value. Including soybean, vulnerability and EVP were positively correlated with the Human Development Index (HDI), Gross Domestic Product (GDP), habitat loss, and farmland size. On the other hand, these key indicators were negatively associated with the human footprint and the proportion of low-income farmers. Excluding soybean, however, vulnerability increased across low-income municipalities, particularly across the Atlantic Forest, Cerrado, and Caatinga, revealing strong biome contrasts. Overall, EVP was consistently reduced in areas under heavier human footprint, suggesting that anthropogenic pressures reduce pollination benefits. Our results also show that soybean cultivation masks underlying social and regional disparities in agricultural vulnerability. Thus, the evaluation of pollination risks can be masked by commodity production. Enhancing ecological processes and promoting diversified, pollinator-friendly agriculture may strengthen food production, particularly for tropical low-income farmers in vulnerable regions, while contributing to ecosystem service provision and human well-being.

## 1. Introduction

Agricultural expansion is one of the main drivers of land degradation [1,2], leading to the loss of natural habitats and biodiversity, consequently disrupting ecosystem dynamics and decreasing their capacity to maintain ecological functions and services [3,4], mainly in tropical regions (e.g., [1]). In tropical developing countries, biodiversity loss and degraded ecosystem functions can be inextricably intertwined with social inequality [5]. This is particularly concerning in Brazil, as more than half of the population (116.8 million people) are under food insecurity or hunger (e.g., [4,6,7]), even though the country is one of the leading food producers [6,8]. In Brazil, as in many other tropical countries, most of the food production for domestic consumption comes from smallholders and family farming [9]. In total, 76.8% of all Brazilian farms belong to family units [10], indicating that decreases in agricultural production, due to pollinator loss, are expected to affect the entire supply chain, from family farmers to large producers, particularly the poor and the rural population [11]. A wide variety of crops cultivated in the tropics depend on pollinators [12,13]. In Latin America, smallholder farmers are responsible for producing more than half of the food consumed by humans [14,15]. Yet, because of the relentless agricultural expansion in the tropics, smallholders are now more vulnerable to declines in animal pollinators, which can aggravate food insecurity [16]. Brazilian agriculture, spanning from smallholders to large-holders, is heavily reliant on biotic pollinators [13,17].

Animal pollination is crucial both as an ecosystem function and as an ecosystem service for people, providing essential inputs to crop yields worldwide [18]. Whether they are cultivated or wild, approximately 90% of all flowering plant species [19] are pollinated by a wide diversity of pollinators, including bees, other insects (e.g., moths, butterflies, wasps, and beetles), bats, birds, and other vertebrates (e.g., [13,19–21]). Natural pollinators ensure or enhance the fruit set, yield, fruit quantity, and quality of over 75% of all global food crops [12,18,20]. Consequently, pollination service provides essential micro- and macronutrients to human health, especially lipids, and vitamins [22–24]. In this context, pollination emerges as a nature contribution to people, as it generates positive benefits for humans through nature [25,26]. Therefore, maintaining the delivery of ecosystem goods has been recognized as imperative for the Sustainable Development Goals [27].

Nevertheless, despite the critical importance of pollination to human well-being, this vital ecosystem service has been severely impacted by human activities on natural ecosystems. Cropland expansion is one of the main drivers of pollinator decline and is still a common strategy to increase crop production, especially in developing countries [28]. Notwithstanding, in both the developed and developing worlds, the area allocated to pollinator-dependent crops has increased faster than that of non-dependent crops [4,29,30]. However, such a trend is largely associated with growing areas of large-scale mechanized monoculture (such as soybean) and, consequently, greater isolation from natural habitats [4,30]. The demand for animal pollination services is rising while pollinator abundance and diversity have been declining [21,29]. In Brazil, for example, a scenario of a pollination crisis could reduce the production of food derived from pollinator-dependent crops by 13.5% – 41.6%, resulting in monetary losses of US$4.86–14.56 billion per year [11]. Losses in pollination services can induce simultaneous degradation of several other ecosystem services [31], and, therefore, cause interlinked poverty spirals and major risks for humankind [32].

As an essential asset in agricultural systems, decreases in pollination services can reduce cropland productivity [30] and economic gains [33,34]. Annually, the economic global input of pollinators to agriculture, inflation-adjusted for 2020, conservatively ranges from US$195 billion to US$387 billion [34], but this value is likely underestimated as economic valuations fail to represent the complex sets of pollinator benefits, including the value of locally grown crops for trade and subsistence, especially in tropical countries [34]. Studies addressing the economic contribution of pollinators overlook the interdependence between biotic pollination and family farming, smallholders, and low-income farmers (but see [16,35–37]. Poverty research does not consider the impacts of pollinator losses and research on the impact of biodiversity loss on poverty rarely reflects pollinators as an essential part of biodiversity [16].

More detailed research on the relationship between pollinator loss and various agricultural systems, considering the spectrum of agricultural income, can provide stronger policy arguments to advocate for pollinator protection [21]. Here, we aimed to explore the complex interactions between environmental, agricultural, and socioeconomic factors, while developing a nationally representative assessment of pollination contribution to agriculture, accounting for rural and socioeconomic diversity. Specifically, we evaluate how the economic value of pollination (EVP) and the vulnerability to pollinator failure correlate with socioeconomic predictors [Gross Domestic Product (GDP), Human Development Index (HDI) and Gini coefficient] and agricultural profile (farm size and farmers income), and how these variables relate to the degree of degradation of administrative jurisdictions. We tested the hypotheses that socioeconomic inequality indicators and agricultural profile influence municipal vulnerability to pollination service loss, specifically, municipal counties with smaller farm sizes, with higher proportion of low-income farmers, with lower HDI and higher Gini coefficient are more vulnerable to losses of pollination services. Additionally, we evaluated whether municipalities exposed to higher EVP tend to have higher GDP and HDI.

## 2. Materials and methods

### 2.1. Agricultural production

We selected the 60 most important agricultural crops in Brazil in terms of total production volume in 2019 [10]. This recent annual harvest (2019) is representative of the country’s typical average agricultural production and the relative contribution of different crops to the overall nutrient supply. These crops comprise almost the entire Brazilian agricultural production. The cultivar list includes Brazilian staple foods (such as rice, beans, and oranges), crops used in livestock feed that result in other staple foods (e.g., eggs, meat and dairy products), and important export commodities (e.g., soybean, sugar cane, cotton, maize, and wheat). We obtained the production value of all crops grown in each of 5,499 Brazilian municipalities in 2019 on the basis of data from the Brazilian Institute of Geography and Statistics [10].

### 2.2. Socioeconomic indicators, farm size, and landholder income

To account for the municipality-scale level of development, we used the human development index (HDI), a widely used indicator that encompasses three key dimensions of human development: living standard, knowledge, and health. To assess the economic aspect, we obtained the GDP of each municipality, and to measure the municipal inequality, we obtained the Gini coefficient of all Brazilian municipalities. All indicators are available at IBGE [10].

To determine a municipal index of farmers’ income, we apply an opportunity cost methodology to classify types of family farmers [38]. Family farming income is categorized into types A, B, C, and D, where type A has the highest income level, while D represents low-income farmers. We then established the municipal index of low-income farmers from the proportion of farmers of type D for each county, available from IBGE [10]. To determine an index of farmland size by municipality, an average of farm size was calculated from the number and total area of rural properties across all Brazilian municipalities, available from IBGE [10].

### 2.3. Financial efficiency of crops

We calculated the financial efficiency of all crops by yield in relation to the total production area, from municipal production data available at IBGE [39]. We then related the financial efficiency of all crops to the dependence of each crop on biotic pollinators.

### 2.4. Land degradation

We accessed data on the cumulative human pressure on the environment as of 2009 at a spatial resolution of ~1 km, for the entire Brazilian territory. This measure of human footprint is based on eight variables, including built-up environments, human population density, electric power infrastructure, croplands, pasture lands, roads, railways, and navigable waterways. The data set was produced by Venter et al. [40] and is available in the Mollweide projection (https://doi.org/10.7927/H46T0JQ4). For data on primary habitat loss, we used Brazil’s annual land use and land cover maps over 36 years (1985–2020) at a 30-m resolution, available from the MapBiomas project (https://mapbiomas.org).

### 2.5. Dependence on pollinators and Economic Value of Pollination (EVP)

Considering that most food crops rely on biotic pollination to a varying extent, we applied the currently most widely accepted standard of relative dependence on pollinators in world agriculture [18,41]. Based on the degree to which crop production and quality in fruits and seeds are associated with animal pollination, crop types examined here were assigned to one of five categories of pollinator dependence (D), adapted from Klein et al. [18] and Gallai et al. [33]: (1) essential (D = 0.95); production is reduced by 90% or more when pollination services are lacking; (2) great (D = 0.65): reduction of 40% to 90%; (3) modest (D* *= 0.25): reduction of 10% to 40%; (4) little (D = 0.05): reduction of 0% to 10%; (5) no differences in yields (D* *= 0) under conditions with and without animal-mediated pollination.

Our database consisted of the production value of leading crops and their respective pollination dependence. We thus calculated, using a bioeconomic approach, the economic contribution of pollination (i.e., the economic value of pollination; EVP) across all municipalities, as well as their overall vulnerability to pollinator declines [33,42]. We adapted the equation proposed by Gallai et al. [33], which estimates the total production value of insect pollination (IPEV), with the variables crop *i*, where *i* ∈ [1; I], in each world region *x*, where *x* ∈ [1; X], quantity produced (Q*ix*) and the dependence ratio of crop *i* on insect pollinators (D*i*) and the price of crop *i* per unit produced in region *x* (P*ix*):


$$\text{IPEV}=\sum {\mathrm{\backslash nolimits}}_{i=\text{1}}^{I}\sum {\mathrm{\backslash nolimits}}_{x=\text{1}}^{X}\left(P_{ix}\;\text{x}\;Q_{ix}\;\text{x}\;D_{i}\right)$$

Instead of using the price of each crop and quantity produced (P*i* × Q*i*), we substituted the *production value* (PV*ix*) available in the IBGE database, which represents the production price of crop (*i*) paid to producers at each municipality (*x*), which we consider, in this case, a more accurate approach:


$$\text{EVP}=\sum {\mathrm{\backslash nolimits}}_{i=\text{1}}^{I}\sum {\mathrm{\backslash nolimits}}_{x=\text{1}}^{X}\left({PV}_{ix}\;\text{x}\;D_{i}\right)$$

### 2.6. Vulnerability to pollinator declines

Based on Gallai et al. [33], vulnerability is defined as the potential production loss attributable to a lack of pollinators, which is calculated as the ratio of EVP to the economic production value of an area (*x*) and can be stated as:


$$\text{Vulnerability}=\frac{\sum_{i=\text{1}}^{I}\sum_{x=\text{1}}^{X}\;\left({PV}_{ix}\;\text{x}\;D_{i}\right)\;}{\sum_{i=\text{1}}^{I}\sum_{x=\text{1}}^{X}\;\left({PV}_{ix}\right)}(\%)$$

### 2.7. Statistical analysis

In this study, municipal counties were adopted as the unit of analysis, totaling 5,499 records. Indicators of crop yield vulnerability to pollinator decline and the economic value of pollination (EVP) across Brazilian municipalities were examined via generalized linear mixed models (GLMMs). Vulnerability to loss of biotic pollination was modeled using a zero-inflated beta regression with logit link (*glmmTMB* package), while EVP was modeled using a Tweedie regression with a log link [43]. Standardized predictors (z-scores) are as follows: farm size (log-transformed), Gini coefficient, Human Development Index (HDI), GDP (log-transformed), the human footprint index, habitat loss (log-transformed), proportion of low-income farmers, and the interaction between low-income farmers and biome. Biome-level differences were evaluated by extracting estimated marginal means with Tukey-adjusted pairwise comparisons for vulnerability (*emmeans* and *multcomp* packages) and by comparing total EVP values across 2000 bootstrap resamplings, from which pairwise differences and *p*-values were obtained. Multicollinearity among predictors was tested using variance inflation factors (VIF, car package), with all values < 3, indicating no major collinearity problems. Subsequently, in order to evaluate fit-quality of selected models, we also assessed model residuals using the DHARMa package, which applies a simulation-based approach to generate scaled (quantile) residuals for fitted generalized mixed models [44]. This diagnostic framework allows for the detection of typical model misspecifications (e.g., deviations from residual uniformity, overdispersion, and residual autocorrelation). To account for potential spatial autocorrelation, Moran’s Eigenvector Maps (MEMs) were incorporated into the models [45]. The MEMs are spatial predictors that maximize spatial autocorrelation measured by Moran’s coefficient. Spatial autocorrelation of residuals was further evaluated using Moran’s *I* index. After adding MEMs, the initially high Moran’s *I* values slightly dropped, suggesting that spatial structure was partially controlled (S1 Table). All statistical analyses were performed using R v. 3.5.3 [46].

## 3. Results

### 3.1. Economic value of pollination and vulnerability at the national level

**Fig 1:**
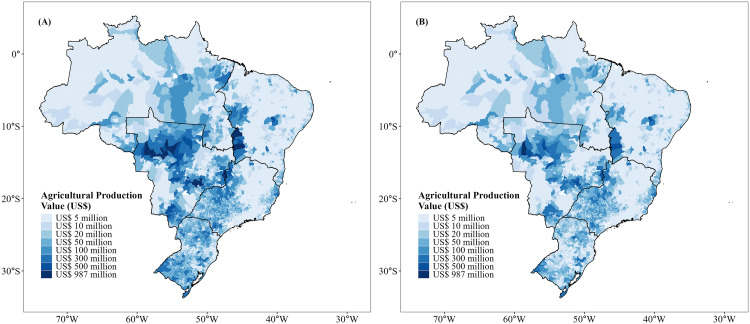
Total agricultural production value of leading crops in the 2019 harvest across all Brazilian municipalities. **(A)** Considering 60 leading crops; and **(B)** Considering 59 leading crops (excluding soybean). Source: IBGE 2021.

**Fig 2:**
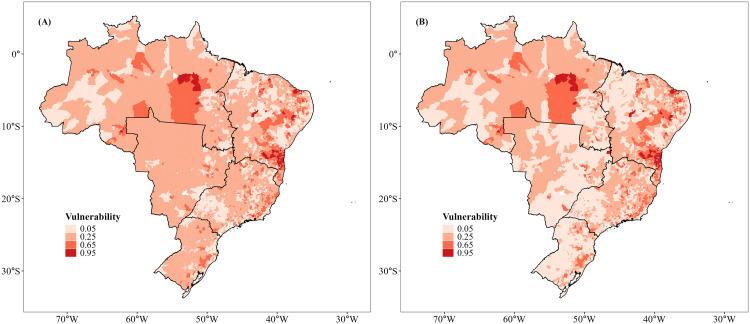
The economic value of pollination in each municipality across Brazil, considering the 2019 harvest. **(A)** Considering 60 leading crops; **(B)** Considering 59 leading crops (excluding soybean). Source: IBGE 2021.

**Fig 3:**
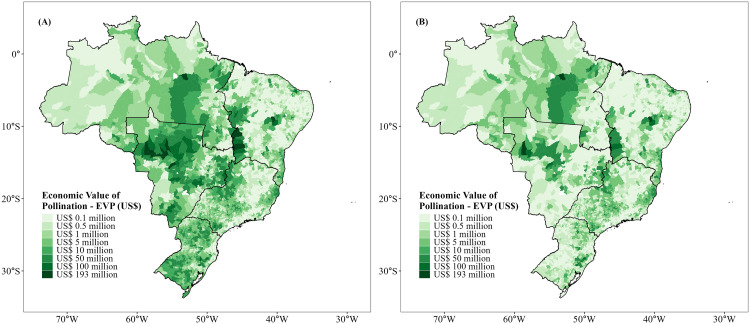
Agricultural vulnerability to pollinator declines in each municipality across Brazil, considering the 2019 harvest. **(A)** Considering 60 leading crops; **(B)** Considering 59 leading crops (excluding soybean). Source: IBGE 2021.

Our results indicate that 35 (58%) of the leading agricultural crops across Brazil are dependent on animal pollination to some degree (Table 1). The spatial patterns of agricultural economic value and the economic value of pollination (Fig 1 and 2) reflected the widespread contribution of pollinators across the country. Agricultural production value, including soybean monoculture (Fig 1A), was spatially concentrated in the Central-West and Southern regions, reflecting the dominance of soybean cultivation in Brazil. When soybean croplands were excluded (Fig 1B), the spatial pattern changed, revealing an even distribution of production values across the Northeast, Southeast, and Northern portions of Brazil. A similar pattern was observed for the economic value of pollination. The inclusion of soybean (Fig 2A) emphasized regions under intensive grain production, particularly the Central-West. By excluding soybean (Fig 2B), there was a strong dependence on diversified agriculture in the Northeast, Southeast, and parts of the Northern region; i.e., soybean thus masked regional vulnerabilities and the economic importance of pollinator-dependent crops at the national scale. Overall, agricultural production in 96.8% of all municipalities was at least partly vulnerable to the loss of pollinators (Fig 3). Considering the agricultural vulnerability to pollinator loss, we observed that, including soybean (Fig 3A), the highest vulnerability was again concentrated in the Central-West and Southern Brazil. Excluding soybean (Fig 3B), however, vulnerability was more evident along the Atlantic coast and in the Northeast.

**Table 1:** The 60 leading crops cultivated in Brazil, the economic value of pollination (EVP) of the harvest in 2019 and the degree of pollination dependence. Information on the degree of cropland dependence on pollinators are sourced from Klein et al. (2007), much of which are reiterated in Roubik (2018)^1^; Giannini et al. (2015)^2^; Campbell et al. (2018)^3^ and Dorneles *et al*. (2013)^4.^

Crop	Latin name	EVP	Pollinator dependence
Soybean	*Glycine max* ((L.) Merr.)	US$7,849,430,000	Modest^1^
Coffee	*Coffea arabica* (L.)	US$1,102,621,625	Modest^1^
Cotton	*Gossypium* spp.	US$999,640,438	Modest^1^
Tomato	*Lycopersicon esculentum* (Mill.)	US$920,893,675	Great^1^
Cocoa	*Theobroma cacao* (L.)	US$597,073,813	Essential^3^
Watermelon	*Citrullus lanatus* (Citrullus)	US$365,402,538	Essential^1^
Apple	*Pyrus malus* (L.)	US$295,120,638	Great^1^
Passion fruit	*Passiflora edulis* (Sims)	US$281,829,375	Essential^1^
Mango	*Mangifera indica* (L.)	US$266,383,325	Great^1^
Assai	*Euterpe oleracea* (Mart.)	US$189,179,938	Modest^1^
Guava	*Psidium guajava* (L.)	US$150,631,650	Great^2^
Melon	*Cucumis melo* (L.)	US$137,436,263	Essential^1^
Orange	*Citrus aurantium* (L.)	US$118,882,875	Little^1^
Bean	*Phaseolus vulgaris* (L.)	US$93,350,700	Little^1^
Peach	*Prunus persica* ((L.) Stokes)	US$62,475,238	Great^1^
Avocado	*Persea americana* (Mill.)	US$58,864,488	Great^1^
Coconut	*Cocos nucifera* (L.)	US$58,102,313	Modest^1^
Cashew nut	*Anacardium occidentale* (L.)	US$24,140,438	Modest^1^
Sunflower	*Helianthus annuus* (L.)	US$23,859,875	Great^2^
Lemon	*Citrus latifolia* (Tanaka)	US$19,636,838	Little^1^
Papaya	*Carica papaya* (L.)	US$13,246,225	Little^1^
Peanut	*Arachis hypogaea* (L.)	US$13,212,813	Little^1^
Mandarin	*Citrus reticulata* (Blanco)	US$12,496,800	Little^1^
Urucum	*Bixa orellana* (L.)	US$11,451,063	Essential^1^
Oil palm	*Elaeis guineensis* (L.)	US$8,033,850	Little^1^
Pear	*Pyrus communis* (L.)	US$6,909,663	Great^1^
Guarana	*Paullinia cupana* (Kunth)	US$6,172,075	Great^1^
Fig	*Ficus carica* (L.)	US$5,516,125	Modest^1^
Palm heart	*Euterpe edulis* (Mart.)	US$3,764,300	Little^3^
Persimmon	*Diospyros kaki* (L.f.)	US$3,680,488	Little^1^
Broad bean	*Vicia faba* (L.)	US$3,637,875	Modest^1^
Castor beans	*Ricinus communis* (L.)	US$2,712,625	Modest^1^
Quince	*Cydonia oblonga* (Mill)	US$191,425	Great^1^
Pea	*Pisum sativum* (L.)	US$145,063	Little^1^
Flax seed	*Linum usitatissimum* (L.)	US$63,075	Little^1^
Pineapple	*Ananas comosus* (L.) Merr.)	0	Non-dependent^1^
Garlic	*Allium sativum* (L.)	0	Non-dependent^1^
Rice	*Oryza* spp.	0	Non-dependent^1^
Oat	*Avena sativa* (L.)	0	Non-dependent^1^
Olive	*Olea europaea* (L.)	0	Non-dependent^1^
Banana	*Musa paradisiaca* (L.)	0	Non-dependent^1^
Sweet potato	*Ipomoea batatas* ((L.) Lam.)	0	Non-dependent^1^
Potato	*Solanum tuberosum* (L.)	0	Non-dependent^1^
Sugar cane	*Saccharum officinarum* (L.)	0	Non-dependent^1^
Onion	*Allium* spp.	0	Non-dependent^1^
Rye	*Secale cereal* (L.)	0	Non-dependent^1^
Barley	*Hordeum vulgare* (L.)	0	Non-dependent^1^
Tea	*Camellia sinensis* ((L.) Kuntze)	0	Non-dependent^1^
Yerba mate	*Ilex paraguariensis* (A. St.-Hil.)	0	Non-dependent^1^
Tobacco	*Nicotiana tabacum* (L.)	0	Non-dependent^1^
Mallow	*Malva sylvestris* (L.)	0	Non-dependent^1^
Cassava	*Manihot esculenta* (Crantz)	0	Non-dependent^1^
Corn	*Zea mays* (L.)	0	Non-dependent^1^
Walnut	*Juglans nigra* (L.)	0	Non-dependent^1^
Black pepper	*Piper nigrum* (L.)	0	Non-dependent^1^
Sisal	*Agave sisalana* (Perrine)	0	Non-dependent^1^
Sorghum	*Sorghum bicolor* (L.) Moench	0	Non-dependent^1^
Wheat	*Triticum* spp.	0	Non-dependent^1^
Triticale	*Tritico secale* Wittmack	0	Non-dependent^1^
Grape	*Vitis* spp.	0	Non-dependent^1^

**Fig 4:**
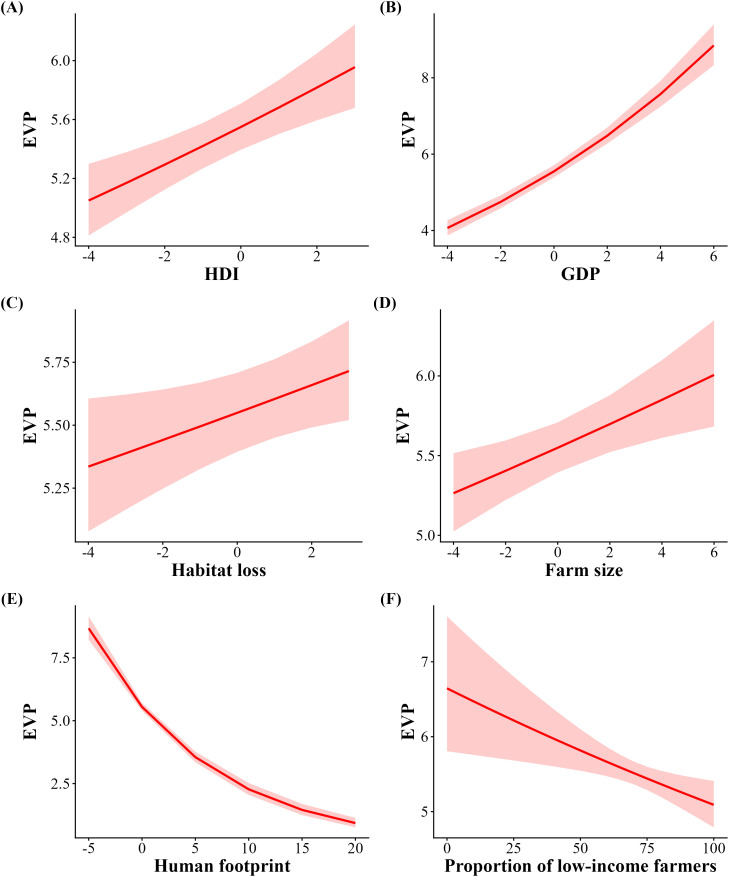
Correlation between the municipal economic value of pollination including soybean and Gross Domestic Product (GDP), Human Development Index (HDI), Habitat loss, Farm size, Human footprint, and Proportion of low-income farmers. Red shading indicates 95% confidence intervals.

**Fig 5:**
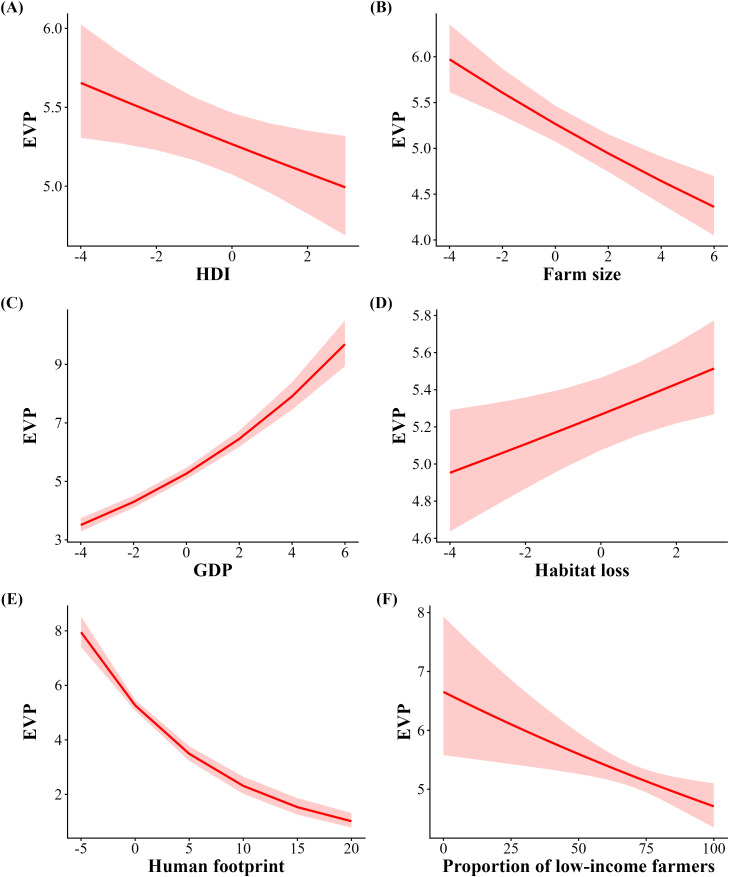
Correlation between the municipal economic value of pollination excluding soybean and Human Development Index (HDI), Gross Domestic Product (GDP), Human footprint, Habitat loss, and Proportion of low-income farmers. Red shading indicates 95% confidence intervals.

The modestly dependent soybean yield (35% of all agricultural production) had the highest EVP, which represented up to US$7.8 billion, followed by coffee (US$1.1 billion, modestly dependent), cotton (~US$1 billion, modestly dependent), tomato (US$0.9 billion, greatly dependent) and cocoa (~US$0.6 billion, essentially dependent) (Table 1). The total economic value of pollination was ~ US$13.8 billion, more than half of which was accounted for by soybean cultivation. This translates into a contrasting economic value of pollination when soybean was either included or excluded from the analysis. With soybean (Table 2), EVP was positively associated with Human Development Index (HDI; Table 2, Fig 4A), Gross Domestic Product (GDP; Table 2, Fig 4B), Habitat loss (Table 2, Fig 4C), and farmland size (Table 2, Fig 4D). In contrast, EVP was negatively related to the human footprint (Table 2, Fig 4E) and proportion of low-income farmers (Table 2, Fig 4F). By excluding soybean (Table 2), patterns shifted substantially. Specifically, HDI and farmland size became negatively related to EVP (Table 2, Fig 5A and B), while GDP (Table 2, Fig 5C) and habitat loss (Table 2, Fig 5D) remained positively associated with EVP. The human footprint index and low-income farmers continued to exhibit a negative association with EVP (Table 2, Fig 5E and F).

**Table 2:** Results of the generalized linear mixed model (GLMM) using a Tweedie distribution (log link). The response variable is the economic value of pollination. Estimates (β), standard errors, z-values, and 95% confidence intervals (CI, low – high) p-values are shown. Significance levels were assessed at a P < 0.05 threshold.

Predictor	β	Std. Error	z-value	p-value	CI (95%)
**Soybean included**					
Gini coefficient	0.006	0.004	1.572	0.116	−0.002–0.015
HDI	0.023	0.005	4.153	**<0.0001**	0.012–0.035
GDP	0.077	0.004	16.225	**<0.0001**	0.068–0.087
Human footprint	−0.089	0.004	−17.933	**<0.0001**	−0.099 – −0.080
Habitat loss	0.009	0.004	2.181	**0.029**	0.001–0.019
Low-income farmers	−0.002	0.0009	−2.801	**0.005**	−0.005 – −0.001
Farm size	0.013	0.004	3.044	**0.002**	0.005–0.022
**Soybean excluded**					
Gini coefficient	−0.002	0.005	−0.493	0.621	−0.014–0.009
HDI	−0.017	0.007	−2.355	**0.018**	−0.033 – −0.003
GDP	0.101	0.006	15.838	**<0.0001**	0.089–0.114
Human footprint	−0.082	0.006	−12.726	**<0.0001**	−0.095 – −0.070
Habitat loss	0.015	0.006	2.529	**0.011**	0.003–0.027
Low-income farmers	−0.003	0.001	−2.781	**0.005**	−0.006 – −0.001
Farm size	−0.031	0.005	−5.400	**<0.0001**	−0.043 – −0.020

**Fig 6:**
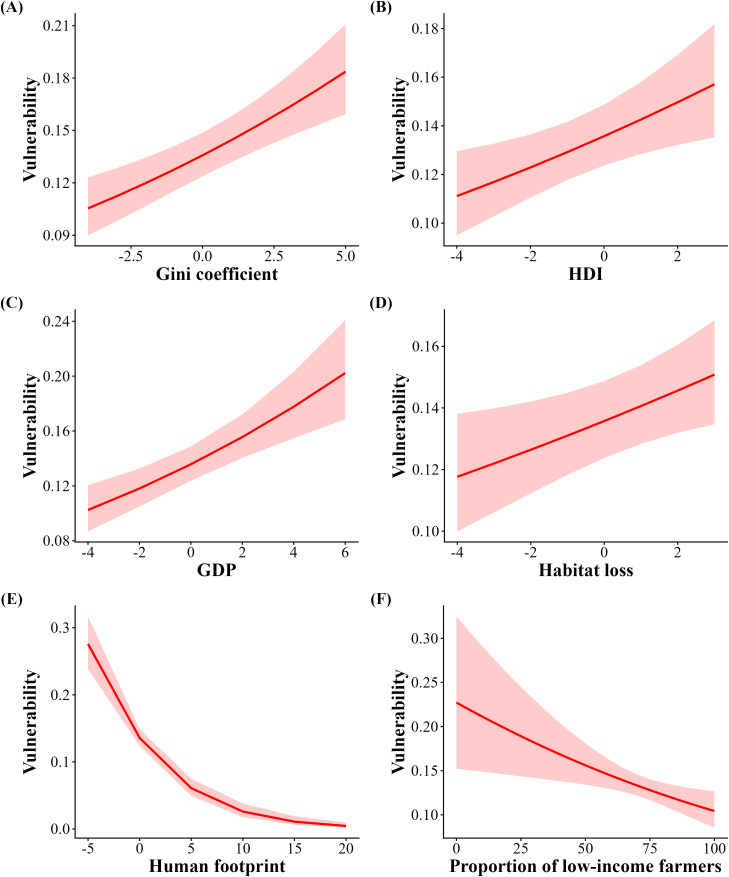
Correlation between municipal scale vulnerability to pollinator declines including soybean cultivation and Human Development Index (HDI), Gross Domestic Product (GDP), Human footprint, Habitat loss, and Proportion of low-income farmers. Red shading indicates 95% confidence intervals.

**Fig 7:**
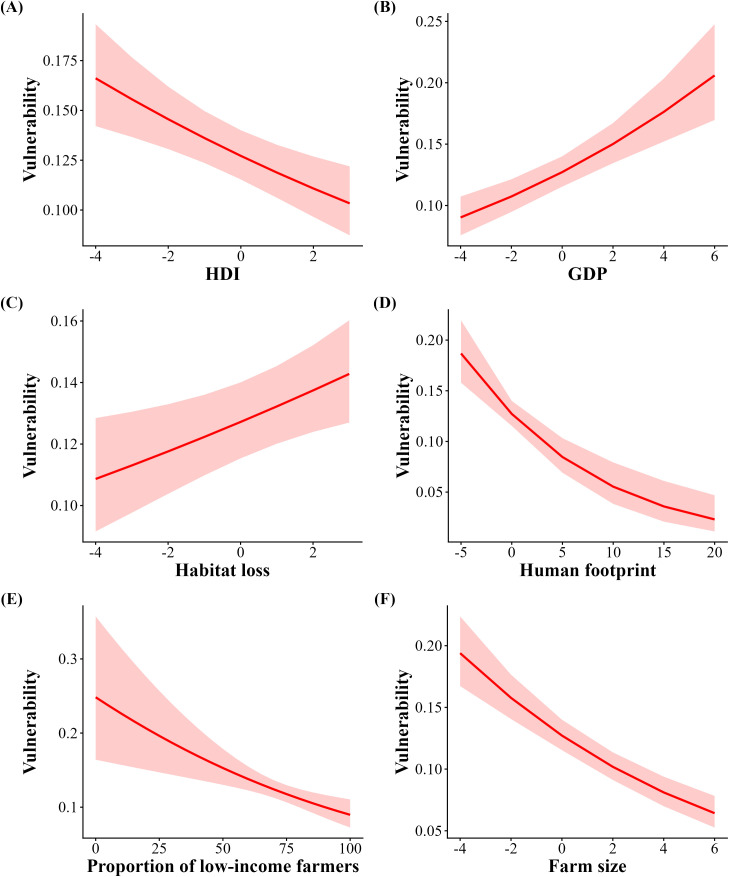
Correlation between municipal scale vulnerability to pollinator declines excluding soybean cultivation and Human Development Index (HDI), Gross Domestic Product (GDP), Human footprint, Habitat loss, and Proportion of low-income farmers. Red shading indicates 95% confidence intervals.

The average vulnerability of Brazilian municipalities was as high as 0.138, indicating that ~14% of the overall agricultural production may be reduced by the loss of pollinators. When soybean was included, vulnerability was positively associated with income inequality (Gini coefficient; Table 3, Fig 6A), HDI (Table 3, Fig 6B), GDP (Table 3, Fig 6C), and rate of habitat loss (Table 3, Fig 6D), while the human footprint (Table 3, Fig 6E) and proportion of low-income farmers (Table 3, Fig 6F) were negatively related to vulnerability. When soybean was excluded, HDI, the human footprint, proportion of low-income farmers, and farm size were negatively associated with vulnerability (Table 3, Figs 7A, D, E and F) while GDP and habitat loss remained positively related (Table 3, Figs 7B and C).

**Table 3:** Results of the generalized linear mixed model (GLMM) using a Tweedie distribution (log link). The response variable is the vulnerability to pollinator decline. Estimates (β), standard errors, z-values, and p-values and 95% confidence intervals (CI, low – high) are shown. Significance levels were assessed at P < 0.05.

Predictor	β	Std. Error	z-value	p-value	CI (95%)
**Soybean included**					
Gini coefficient	0.071	0.015	4.592	**<0.0001**	0.041–0.103
HDI	0.057	0.020	2.780	**0.005**	0.017–0.097
GDP	0.079	0.017	4.523	**<0.0001**	0.045–0.097
Human footprint	−0.177	0.018	−9.585	**<0.0001**	−0.213 – −0.141
Habitat loss	0.040	0.016	2.440	**0.014**	0.008–0.074
Low-income farmers	−0.009	0.003	−2.693	**0.007**	−0.016 – −0.003
Farm size	−0.017	0.015	−1.151	0.249	−0.048–0.013
**Soybean excluded**					
Gini coefficient	0.023	0.016	1.435	0.151	−0.009–0.057
HDI	−0.078	0.021	−3.572	**<0.0001**	−0.121 – −0.035
GDP	0.096	0.018	5.120	**<0.0001**	0.059–0.133
Human footprint	−0.091	0.018	−4.980	**<0.0001**	−0.127 – −0.055
Habitat loss	0.044	0.017	2.599	**0.009**	0.011–0.078
Low-income farmers	−0.012	0.003	−3.320	**0.0008**	−0.019 – −0.005
Farm size	−0.125	0.016	−7.588	**<0.0001**	−0.158 – −0.093

### 3.2. Economic value of pollination and vulnerability at the biome scale

Contrasting trends in EVP and vulnerability across biomes emerged, regardless of whether or not soybean cultivation was included. Including soybean, most biomes exhibited negative correlations between poverty and EVP, especially the Amazon, the Atlantic Forest, and the Pantanal wetlands, where the incidence of low-income farmers increased and EVP sharply decreased (S1 Fig). On the other hand, the Caatinga and the Pampa exhibited positive correlations, suggesting that the relative importance of pollinators rises for low-income counties in these biomes. For example, in the Caatinga, greater vulnerability under socioeconomic constraints resulted from the predominance of pollinator-dependent crops (S1 Fig). When soybean was included in the production system, the Cerrado’s response was almost flat, indicating that the income structure had less impact on EVP (S1 Fig). When soybean was excluded, a more heterogeneous pattern emerged across biomes. Although the slopes were even steeper, the Amazon and the Atlantic Forest still showed noticeable declines in EVP as poverty increased (S2 Fig). Previously exhibiting minimal fluctuation, the Cerrado now had a clear decreasing trend, consistent with the trends observed in other biomes (S2 Fig). In contrast to the model including soybean, the Pantanal showed a significantly flatter relationship, whereas the Caatinga and Pampa maintained positive relationships (S2 Fig). These changes resulted from the fact that soybean is a partially pollinator-dependent crop and it is highly concentrated in particular regions.

The interaction between low-income farmers and biomes indicated that when soybean was included, most biomes exhibited gentle slopes, with only the Pampa and Caatinga showing stronger increases in vulnerability as the proportion of low-income farmers increased (S1 Fig). In contrast, when soybean was excluded, the biome-specific responses became much clearer. The Amazon and Pantanal exhibited decreasing vulnerability with higher prevalence of low-income farmers (S2 Fig). In contrast, the Atlantic Forest, Cerrado, Caatinga, and Pampa presented a pronounced positive relationship, with vulnerability sharply increasing as the proportion of low-income farmers increased (S2 Fig).

**Fig 8:**
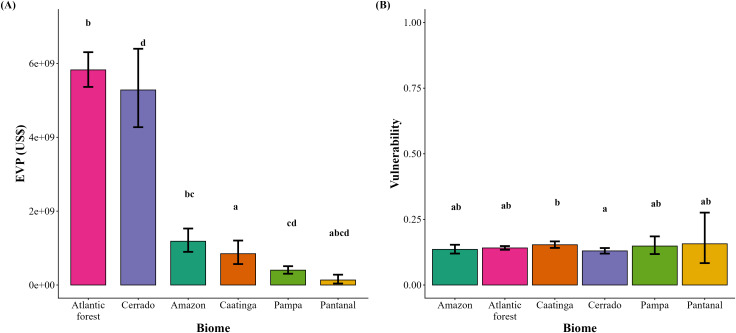
Economic value of pollination (EVP) (A) and vulnerability to pollinator declines (B) across Brazilian biomes including soybean. Bars indicate mean ± SE. Different letters above bars represent significant differences among biomes.

**Fig 9:**
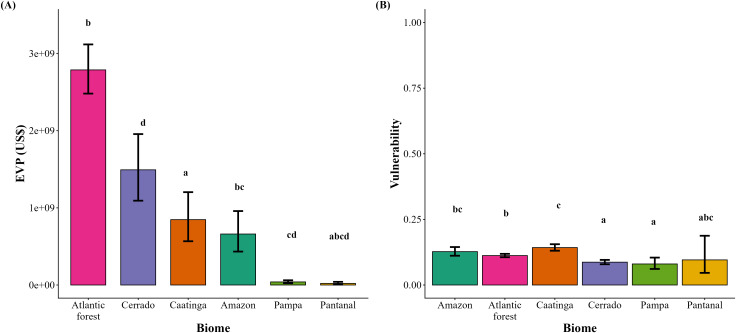
Economic value of pollination (EVP) (A) and vulnerability to pollinator declines (B) across Brazilian biomes excluding soybean. Bars indicate mean ± SE. Different letters above bars represent significant differences among biomes.

In general, the economic value of pollination in Brazil was concentrated in the Atlantic Forest and Cerrado. Nevertheless, when soybean was excluded, the EVP declined to approximately US$6.0 billion (Table 4). EVP varied significantly across biomes, with more striking differences when soybean was excluded from the analysis. Specifically, when soybean was included, the highest EVP was concentrated in the Atlantic Forest and the Cerrado, which did not differ significantly from each other and together represented the largest fraction of the overall value across all biomes (~US$11.2 billion, 81.1% of the total EVP; Table 4, Fig 8A). The Amazon and Caatinga supported intermediate EVP values, but the Amazon exhibited a significantly higher EVP than that observed in the Caatinga (Fig 8A). Meanwhile, the Pampa grasslands and the Pantanal wetlands supported the lowest scores (Fig 8A). In contrast, when soybean was excluded from the estimates, the Atlantic Forest clearly dominated, showing significantly higher EVP than all other biomes (~US$2.8 billion, equivalent to approximately 47% of the total EVP; Table 4, Fig 9A), followed by the Cerrado with considerably lower values (Fig 9A). Under this scenario, the Caatinga and Amazon supported intermediate values, but EVP was significantly higher in the Caatinga compared to the Amazon (Fig 9A). The Pampa and Pantanal presented only marginal values and did not differ from each other, although the Pantanal partially overlapped with some of the intermediate biomes (Fig 9A). We can therefore observe that soybean cultivation substantially increased the EVP in the Cerrado, bringing it to the same level as the Atlantic Forest. Conversely, since the EVP in the Caatinga did not increase with soybean, its relative importance increased, surpassing the Amazon when soybean was excluded, although their absolute values remained similar.

**Table 4:** Estimated total production value, economic value of pollination (EVP) for the entire country and within each biome domain. Values refer to the leading crops in the 2019 harvest. ^1^Source: IBGE.

Location	Production value^1^	Economic value of pollination (EVP)
**Soybean included**		
**Brazil**	~US$90 billion	~US$13.8 billion
**Biome**		
Amazon	~US$6.1 billion	~US$1.2 billion
Atlantic Forest	~US$46 billion	~US$5.9 billion
Caatinga	~US$3.2 billion	~US$0.9 billion
Cerrado	~US$31 billion	~US$5.3 billion
Pampa	~US$3.4 billion	~US$0.4 billion
Pantanal	~US$0.8 billion	~US$0.1 billion
**Soybean excluded**		
**Brazil**	~US$58 billion	~US$6.0 billion
**Biome**		
Amazon	~US$4.0 billion	~US$0.7 billion
Atlantic Forest	~US$33 billion	~US$2.8 billion
Caatinga	~US$3.2 billion	~US$0.9 billion
Cerrado	~US$15 billion	~US$1.5 billion
Pampa	~US$1.9 billion	~US$0.05 billion
Pantanal	~US$0.3 billion	~US$0.03 billion

Vulnerability to pollinator loss across all Brazilian biomes was relatively low and homogeneous when soybean was included in the analysis. The Caatinga presented the highest vulnerability values, and significantly higher than the Cerrado, which presented the lowest scores (Fig 8B). In contrast, the Amazon, Atlantic Forest, Pampa, and Pantanal occupied intermediate positions lacking significant differences (Fig 8B). The vulnerability pattern shifted and the underlying biome disparities were more pronounced after soybean was removed. The Amazon exhibited an increase in vulnerability under this scenario, displaying intermediate values, while the Caatinga retained the highest vulnerability, far above all other biomes (Fig 9B). The Atlantic Forest and Pantanal also exhibited intermediate vulnerability, although the latter showed considerable variation and partially overlapped with the Caatinga and Cerrado. The Cerrado and Pampa were consistently the least vulnerable, did not differ from each other, but differed significantly from the Caatinga. Therefore, it was possible to detect that soybean reduced the vulnerability contrast between biomes, but its exclusion revealed a clearer gradient.

## 4. Discussion

Our results indicate that cropland production in the world’s leading tropical agricultural country depends on biotic pollination to varying degrees. Most of the ~ 5,500 Brazilian municipal counties (96.8%) are, to some extent, vulnerable to pollination failure, which is related to factors such as farmland size, farmer income, level of habitat loss, income inequality, and other socioeconomic indicators. Around 58% of all Brazil’s leading farmland crops rely on animal pollination, with key cultivars such as soybean, coffee, cotton, tomato, and cocoa accounting for 84% of the overall agricultural economic value linked to pollination. Although representing 35% of total production, soybean cultivation accounts for more than half of the total economic value of Brazilian agricultural production. Soybean croplands strongly shape the spatial and socioeconomic patterns of the economic value of pollination and crop vulnerability in Brazil. Soybean homogenizes the responses at the biome-level, while its exclusion amounted to positive associations in the Atlantic Forest, Cerrado, and Caatinga. The relationship between vulnerability and low-income farmers also varies across Brazilian biomes. Thus, patterns of vulnerability to pollinator loss are determined by pollinator dependence and crop diversification across biomes. In light of these challenges, we discuss how enhancing agricultural practices and ecological processes may significantly improve food security, especially for low-income farmers in vulnerable areas.

Our findings reinforce the notion that losses in ecosystem services, measured here by the expected yield losses from biotic pollinator failure, exert more pronounced consequences in an already vulnerable group of rural producers [47]. For instance, smallholder farmers in Madagascar frequently face food production risks, such as pest damage and crop losses, which result in direct negative impacts on income and livelihoods, highlighting the urgent need to reduce future vulnerability to high-risk exposure [48]. In Brazil, subsistence consumption of edible fruits by low-income communities in the Caatinga dry forest is negatively impacted by chronic anthropogenic disturbances and increased aridity [49]. In this context, it is important to understand how losses in ecosystem services will affect the food systems and agricultural yields of smallholders, often represented by the low-income rural poor who subsidise their livelihoods with forest resources. Our results provide empirical evidence for this concern: when soybean was removed from the analysis, municipalities containing higher percentages of low-income farmers showed greater vulnerability to pollinator losses, especially in biomes like the Atlantic Forest, Cerrado, and the Caatinga. Conversely, the dominance of soybean in central-western Brazil obscured such a socioeconomic vulnerability, resulting in weak correlations at the biome level. This contrast reveals that soybean production strongly homogenizes the national pattern, masking the elevated risks faced by smallholders in other regions. These findings (1) call attention to the role major crops can play in relation to vulnerability assessments, and (2) align with broader evidence that smallholders are disproportionately exposed to losses of ecosystem services, as their limited adaptive capacity, small economies of scale, reliance on diversified pollinator-dependent crops, and socioeconomic fragility increase their vulnerability to global change [47,48,50]. In general, low-income smallholders rely on agriculture for subsistence, so that reduced agricultural productivity can have severe impacts on food security and family income [51]. Our findings demonstrate that the interaction between rural poverty and pollination dependence amplifies agricultural vulnerability, and that this interaction, rather than uniform, varies markedly across Brazil’s phytogeographic biomes. This indicates that soybean masks socioecological inequalities and that smallholder vulnerability carries a strong structural component.

The spatial and socioeconomic heterogeneity observed in our results is largely shaped by the central role of soybean in the Brazilian national agricultural economy. Soybean tend to homogenize patterns, shifting the EVP and vulnerability to regions characterized by high mechanized production, which explains the positive association between EVP and variables such as HDI, GDP, habitat loss, and farm landholding size. Higher-income farmers typically embrace modern conventional agriculture, which is centered on intense use of synthetic fertilizers and pesticides, as well as mechanized monoculture production [52,53]. Conventional agriculture is widely known to be one of the main drivers of habitat degradation, which in turn reduces native biodiversity (e.g., [1]). As a consequence, floral diversity and resource availability for pollinators are reportedly decreased (e.g., [6,54]). This contrasts with smallholder farmers, who generally maintain organic or diversified agricultural practices, such as the use of agroforestry systems [55,56]. These systems are widely recognized for supporting higher agricultural diversity, conserving natural habitats, enhancing biodiversity, and promoting pollinator assemblages, thus contributing to the delivery of many ecological services, including pollination [56]. Smallholders are typically low-income farmers and their production is based on family farming, mainly food crops for subsistence [55–62]. Therefore, differences in agricultural practices adopted by farmers of different income levels are reflected in the delivery of ecosystem services, such as pollination. In this sense, the socioeconomic context influences the capacity for sustainable management and the conservation of suitable habitats available for pollinators. Thus, socioeconomic inequality translates into ecological inequality, with wealthier farmers driving habitat degradation (e.g., [47]), while a disproportionate number of poorer farmers depending more directly on ecosystem services but being impacted by habitat loss and degradation of ecosystem functioning [5]. Because they lack institutional support and are less resilient, individuals who are in vulnerable situations are disproportionately impacted [63]. In order to develop successful strategies that balance agricultural production with biodiversity protection, it is essential to incorporate socioeconomic and ecological factors into decision-making.

The Atlantic Forest and Cerrado domains had the highest EVP values, while the Caatinga and Amazonia grouped the most vulnerable municipalities. The contrasts between biomes allow us to interpret certain trends. Despite the additional economic importance, the combination of severe habitat fragmentation, family farming, and crop diversity results in high regional exposure to pollinator declines. Tropical forests are critically threatened by agricultural frontier expansion, mainly dominated by mechanized monocultures such as soybeans, sugar cane, and palm oil [1,4]. In Brazil, soybean cropland expansion alone accounts for a significant fraction of deforestation in the Atlantic Forest, Cerrado, Pampa, and Amazonian biomes [13,64–66]. However, 70% of all soybean plantations in Brazil are in the Cerrado and/or the Amazon [65]. In the Amazon, small-scale farmers depend on agricultural products as their primary source of income, but they continue to face major challenges such as access to markets and land degradation, especially in agricultural frontier areas [67]. The Amazon shows intermediate EVP values and high internal heterogeneity: in agricultural frontier areas, the expansion of soybeans and other monocultures increases habitat loss and puts pressure on family systems, while in rural areas that still retain much forest cover, dependence on pollination can be high locally. Therefore, excluding soybeans, vulnerability in the Amazon tends to increase and reveal hidden inequalities in pollination benefits. The Caatinga domain hosts a population of c. 29 million people who often subsist on natural resources, with family farming being prevalent in local communities [68]. However, the Caatinga has been greatly degraded by chronic disturbances and increased aridity, which could threaten the food systems in the Caatinga dry forest, including the production of edible fruits [49]. The pattern of low EVP in the Caatinga can be explained by the prevalence of low-income smallholders who largely cultivate subsistence crops. On the other hand, the Atlantic Forest had the highest EVP but concentrated municipalities containing smaller and more vulnerable farms. Family farming of food crops in this domain feeds a large fraction of Brazil’s population [69], which can explain the higher EVP values. However, the Atlantic Forest has been historically threatened by habitat loss and fragmentation, which results in biodiversity loss and lower flows of ecosystem services in small farms adjacent to natural habitat patches [70–72]. Therefore, we highlight that smallholders and low-income farmers are more vulnerable not only to socioeconomic factors but also to biotic pollination loss, which can severely reduce yield production and threaten food security.

Finally, we seek to strengthen an emerging discussion on enhancing agricultural yields for smallholders and low-income farmers in Brazil. Mitigation strategies that could combine the maintenance of natural forests, native biodiversity, and agricultural production within the same landscape could reduce the vulnerability of small farmers to pollinator losses. Food security depends on stable agricultural production, and ecological intensification can emerge as a nature-based solution to safeguard or restore pollination services and promote food security by reducing the land use footprint by sparing adjacent patches of natural habitats [54,73]. Our results show that soybean expansion homogenizes nation-wide patterns by masking regional vulnerabilities to pollinator loss, highlighting the need for policies that are not only commodity-oriented but also context-dependent for low-income farmers, i.e., considering the realities of smallholder farmers in accordance with the biome/region they live in. However, amid the growing pressures of global environmental change, it is important to highlight that habitat loss is not the only driver of negative impacts on the delivery of pollination services, but also that the interactive effects of climate change must be integrated into approaches. For example, seeking to understand how rising temperatures and changing precipitation regimes alter the survival, phenology, and spatial distribution of plants and pollinators, which consequently leads to frequent mismatches in plant-pollinator interactions and negatively affects the effectiveness of pollination services in agricultural landscapes (e.g., [74]). Furthermore, in many cases, climate change can exacerbate the impacts of natural habitat fragmentation and degradation. Integrating these drivers into land-use planning is particularly urgent in Brazil, where regional contrasts in pollination vulnerability are profound and where poor farmers disproportionately rely on ecosystem services for their livelihoods. Therefore, future researchers and decision-makers should consider climate change projections as a key factor in developing effective strategies for land-use planning and biodiversity conservation. This will enable policies that promote sufficient levels of retention of pollinator habitats. This can effectively support smallholder agricultural production, creating more ecologically sustainable and resilient agricultural systems in the face of global environmental change, capable of sustaining key ecosystem services, such as pollination. This can ensure that ecosystem services are maintained not only for export-oriented commodity agriculture but also for diverse local food systems. It is important to emphasize that the vulnerability of smallholder farmers is structural, stemming from historically persistent socioeconomic inequalities, limited adaptive capacity, and a strong reliance on pollinator-dependent crops. These structural factors must therefore be assessed for the resilience of agricultural production. Our findings reinforce the notion that ecological strategies alone are insufficient to reduce agricultural vulnerability if underlying social disparities cannot be simultaneously reduced. Therefore, by considering these factors, agricultural landscapes can become more socioecologically sustainable, supporting key ecosystem services such as pollination.

## Supporting information

S1 Table Model selection results for the Economic Value of Pollination (EVP) and Vulnerability to Pollinator Decline models based on the inclusion of Moran’s Eigenvector Maps (MEMs). (PDF)

S1 Fig Relationship between the proportion of low-income farmers and (A) the economic value of pollination (EVP) and (B) the vulnerability associated with pollinator dependence across six Brazilian biomes, with soybean included. Colored lines represent fitted trends for each biome.
(TIFF)

S2 Fig Relationship between the proportion of low-income farmers and (A) the economic value of pollination (EVP) and (B) the vulnerability associated with pollinator dependence across six Brazilian biomes, with soybean excluded. Colored lines represent fitted trends for each biome.
(TIFF)
