# Supplementary material for: Yield vulnerability of low-income smallholders to pollinator declines in Brazil is biome-dependent
Source: PLoS One. 2025 Nov 25;20(11):e0337328. doi: 10.1371/journal.pone.0337328 (PMC12646406; doi:10.1371/journal.pone.0337328)
Supplement: S1 Table — (PDF) [file pone.0337328.s001.pdf]

SUPPORTING INFORMATION

Yield vulnerability of low-income smallholders to pollinator declines in Brazil is biome-dependent

Willams Oliveira[a], Rafaella G. Porto[b], Oswaldo Cruz-Neto[a], Marcelo Tabarelli[a], Blandina F. Viana[c], Carlos A. Peres[d,e,*], and Ariadna V. Lopes[a,*]

[a] Departamento de Botânica, Universidade Federal de Pernambuco, Recife, Pernambuco, 50372-970, Brazil

[b] Programa de Pós-Graduação em Biologia Vegetal, Departamento de Botânica, Universidade Federal de Pernambuco, Recife, Pernambuco, 50372-970, Brazil

[c]Instituto de Biologia, Universidade Federal da Bahia, Salvador, Bahia, 40170-210, Brazil

[d]School of Environmental Sciences, University of East Anglia, Norwich Research Park, Norwich NR4 7TJ, UK

[e] Instituto Juruá, Manaus, Brazil

*Corresponding authors e-mail: c.peres@uea.ac.uk (C.A. Peres) and ariadna.lopes@ufpe.br (A.V. Lopes)

ORCID

W. Oliveira - 0000-0001-8345-7986

R.G. Porto – 0000-0002-4491-6825

O. Cruz-Neto - 0000-0002-6625-7568

M. Tabarelli - 0000-0001-7573-7216

B.F. Viana - 0000-0002-4924-1257

C.A. Peres - 0000-0002-1588-8765

A.V. Lopes – 0000-0001-5750-5913

Table S1. Model selection results for the Economic Value of Pollination (EVP) and Vulnerability to Pollinator Decline models based on the inclusion of Moran's Eigenvector Maps (MEMs).

Model ID	Number of MEMs	AIC	Moran's *I*
**Economic Value of Pollination**			
M0	Base model (0 MEMs)	9412.7	0.426
M1	5 MEMs	9395.8	0.411
M2	10 MEMs	9389.1	0.398
M3*	15 MEMs	9387.6	0.392
M4	20 MEMs	9387.5	0.391
**Vulnerability to Pollinator Decline**			
M0	Base model (0 MEMs)	6128.4	0.509
M1	5 MEMs	6102.9	0.487
M2	10 MEMs	6098.6	0.468
M3*	15 MEMs	6097.3	0.460
M4	20 MEMs	6098.0	0.459

*We selected the model M3, with the inclusion of 15 MEMs, showing the lowest AIC and a substantial reduction in residual spatial autocorrelation (Moran's $I = 0.39$ for EVP and 0.46 for vulnerability). The selected models (M3) were therefore used for all subsequent statistical inferences.
