# Supplementary figures and images for: Yield vulnerability of low-income smallholders to pollinator declines in Brazil is biome-dependent
Source: PLoS One. 2025 Nov 25;20(11):e0337328. doi: 10.1371/journal.pone.0337328 (PMC12646406; doi:10.1371/journal.pone.0337328)

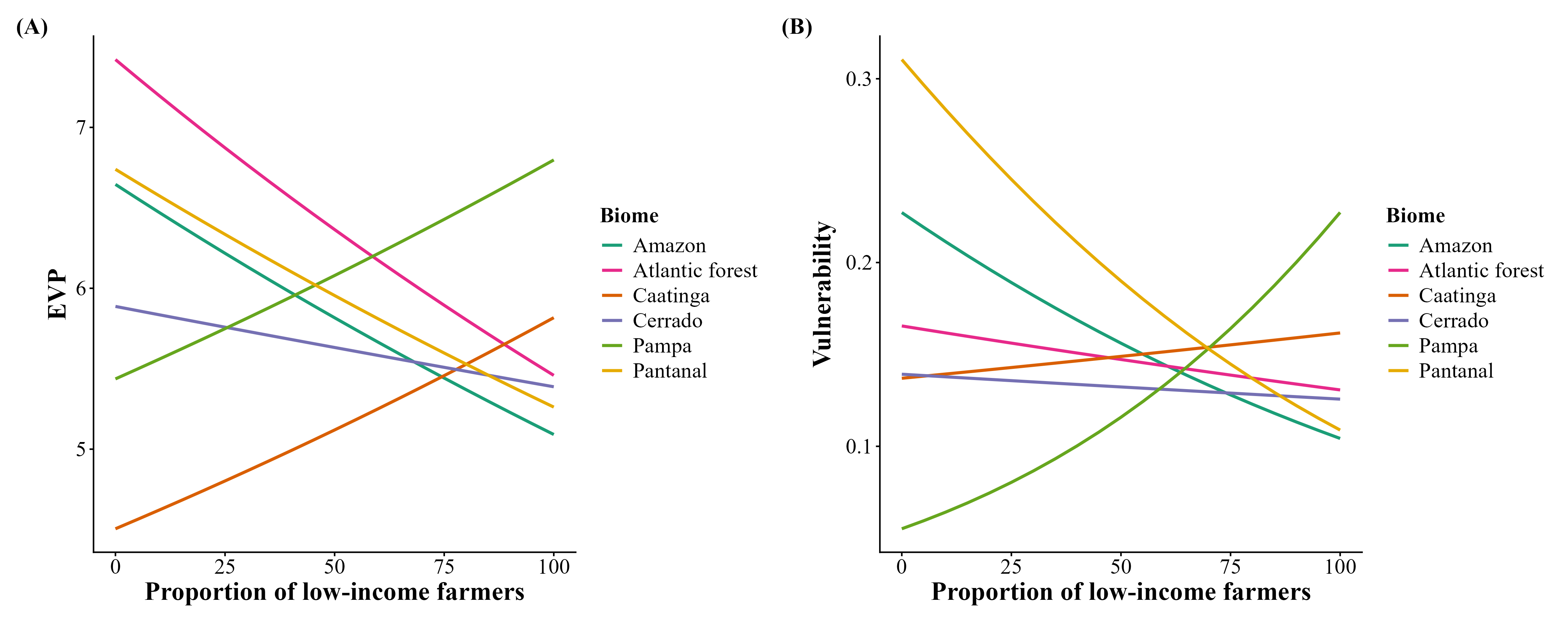

Supplement: S1 Fig — Colored lines represent fitted trends for each biome. (TIFF) [file pone.0337328.s002.tiff]

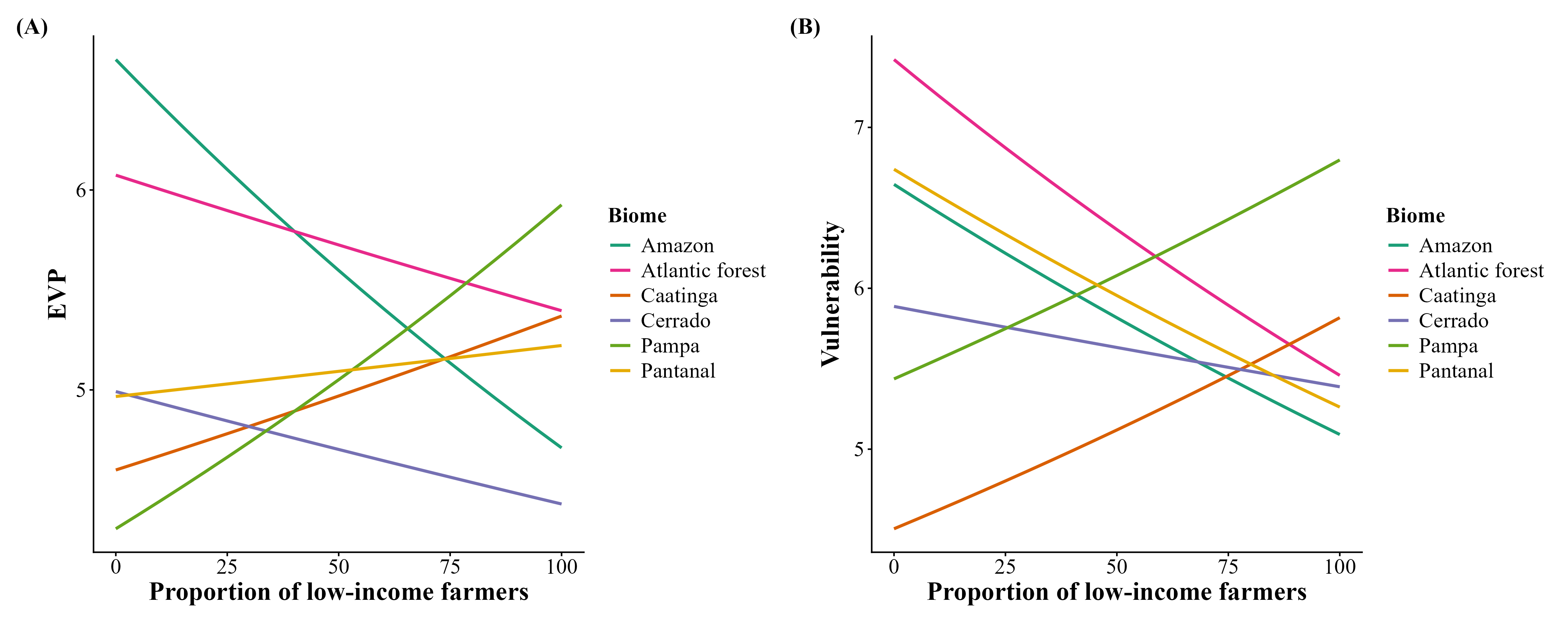

Supplement: S2 Fig — Colored lines represent fitted trends for each biome. (TIFF) [file pone.0337328.s003.tiff]
